# Supplementary material for: Reference-point centering and range-adaptation enhance human reinforcement learning at the cost of irrational preferences
Source: Nat Commun. 2018 Oct 29;9:4503. doi: 10.1038/s41467-018-06781-2 (PMC6206161; doi:10.1038/s41467-018-06781-2)
Supplement: Supplementary file 1 — Supplementary Information [file 41467_2018_6781_MOESM1_ESM.pdf]

Supplementary Information

Reference-point centering and range-adaptation enhance human reinforcement learning at the cost of irrational preferences

Bavard et al.

Supplementary Methods

Model simulations of the POLICY and the UTILITY models

We analyzed the generative performances of the POLICY model: similarly to the RELATIVE model, the POLICY model underestimates the difference between the big and the small magnitude contexts (simulations vs. data, $T(59)=2.9$, $P<0.006$). When considering the transfer test, the POLICY model predicts a linear pattern, because, despite the normalization process within the softmax function, option values remain encoded in an absolute scale. Paradoxically, whereas in the learning sessions the POLICY model predicts a behavior compatible with the RELATIVE model (i.e., no magnitude effect), in the transfer test it predicts a behavior consistent with the ABSOLUTE model (i.e., no value inversion)(**Supplementary Fig. 1 a-c**).

We also analyzed the generative performances of the UTILITY model: similarly to the HYBRID model, the UTILITY model is able to perfectly capture the size of the magnitude effect in the learning sessions (simulation vs. data, $T(59)=0.2$, $P>0.80$). Accordingly, the quality of fit (BIC) difference between these two models was not different when considering the learning sessions alone (HYB vs. UTY, $T(59)=0.2$, $P>0.84$, **Table 3**). However, when considering the transfer test, the UTILITY model unsurprisingly also predicted linear patterns (similar to the ABSOLUTE model), and failed to predict the value inversion between the intermediate options (**Supplementary Fig. 1 d-f**). Accordingly, the quality of fit (BIC) difference between the HYBRID and the UTILITY models was significantly different when considering the transfer sessions alone (HYB vs. UTY, $T(59)=3.3$, $P<0.002$, **Table 3**) (**Supplementary Fig. 1 d-f**).

Additional model comparison: the SEPARATE and the ABS-AC models

The fourth model, referred to as the SEPARATE model, encodes range adaptation and reference-point dependence separately with 2 respective additional free parameters ρ and π. The model describes an absolute value encoding when both parameters are set to 0 and a relative value encoding when both parameters are set to 1 :

$$R_{\text{SEP},t} = (1-\rho) * R_{\text{ABS},t} + \rho * \frac{R_{\text{ABS},t}}{|V_t(s)|} + \pi * \max\left\{0, \frac{-V_t(s)}{|V_t(s)|}\right\}$$

We analyzed the generative performances of the SEPARATE model, which encodes range adaptation and reference-point dependence separately. Coherently, the model behaves similarly to the HYBRID model and captures both the magnitude effect in the learning sessions (simulation vs. data, $T(59)=1.3$, $P>0.18$) and the behavioral patterns when considering the transfer test (**Supplementary Fig. 1 g-i**). However, by increasing its complexity with two additional free parameters, the quality of fit (BIC) difference between the HYBRID and the SEPARATE model was significantly different (HYB vs. SEP $T(59)=5.42$, $P<0.0001$, **Supplementary Table 1**) in favor of the HYBRID model. In addition, we retrieved a significant correlation between the ρ and the π parameter ($R=0.31$, $P<0.02$), partially explaining the fact that a model with the two processes governed by only one parameter is more parcimonious.

We considered a fifth model, referred to as the ABS-AC model, is a mixture between a standard Q-learning algorithm (similar to the ABSOLUTE model) and an actor-critic algorithm[1]. State values, changing over trials, are updated as a function of prediction errors using the delta-rule, such as Q-values in the ABSOLUTE model. Prediction errors in the critic are also used to adjust weights in the actor. Then "hybrid" Q-values are computed and an additional weighting free parameters makes the balance between the two mechanisms :

$$Q_{\text{A-AC},t}(s,a) = w_{\text{A-AC}} * Q_{\text{ABS},t}(s,a) + (1-w_{\text{A-AC}}) * Q_{\text{AC},t}(s,a)$$

with Q_{ABS} the option value updated with the ABSOLUTE (Q-learning) value encoding and Q_{AC} the actor-critic option value updated as follows : $Q_{\text{AC}}(s,a) \longleftarrow Q_{\text{AC}}(s,a) + \alpha_{AC} * (R_{\text{ABS}} - V(s))$, with $V(s)$ the state value at each trial. Action choices are computed using a softmax decision rule, by replacing individual contributions of each model by the mixture value.
To understand why relative model comparison favours the HYBRID model, we analyzed the generative performances of the ABS-AC model: the model doesn't perform as well as participants in the big magnitude context. As a result, it overestimates the difference of performance between magnitude contexts

in the learning phase and fails to match the global performance level. When extrapolating options the transfer test, the model doesn't successfully capture the value inversion and predicts a behavior consistent with absolute value encoding (**Supplementary Fig. 1 j-l**). Accordingly, the quality of fit (BIC) difference between the HYBRID and the ABS-AC models was significantly different (HYB vs. A-AC T(59)=4.80, P<0.0001, **Supplementary Table 1**).

Supplementary References

[1] Gold, J. M. et al. Negative symptoms and the failure to represent the expected reward value of actions: behavioral and computational modeling evidence. *Arch. Gen. Psychiatry* 69, 129–138 (2012).

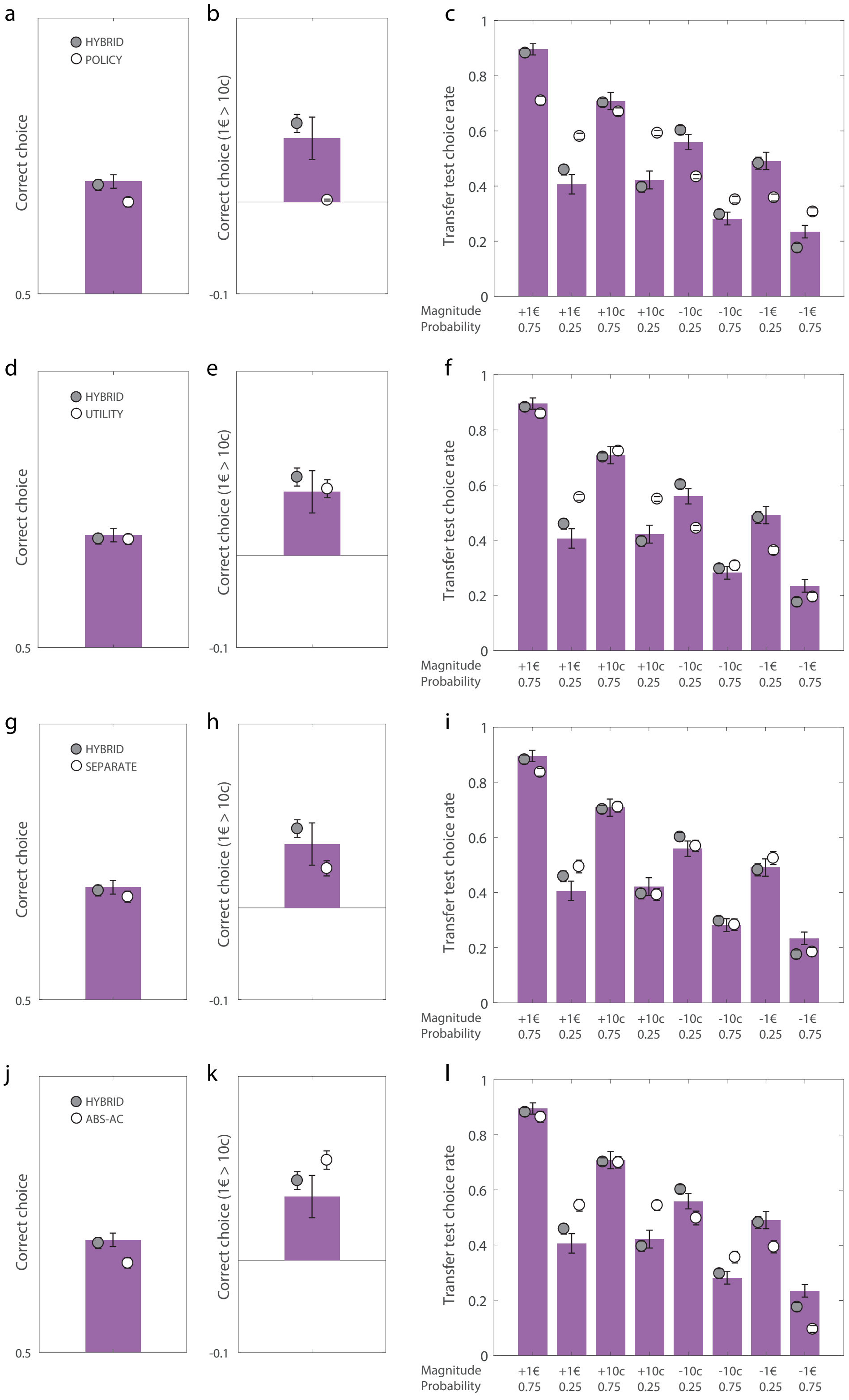

Supplementary Figure 1: Behavioral results and model simulations of Experiment 1 and Experiment 2 pooled together. **a, d, g, j** Correct choice rate during the learning sessions. **b, e, h, k** Big magnitude context's minus small magnitude context's correct choice rate during the learning sessions.**c, f, i, l** Choice rate in the transfer test. Colored bars represent the actual data; grey dots (HYBRID) and white dots represent the model-simulated data; error bars represent s.e.m.

	Experiment 1 (N=20)			Experiment 2 (N=40)			Both experiments (N=60)		
	Learning sessions (nt=160)	Transfer test (nt=112)	Both (nt=272)	Learning sessions (nt=160)	Transfer test (nt=112)	Both (nt=272)	Learning sessions (nt=160)	Transfer test (nt=112)	Both (nt=272)
HYBRID (df=3/4)	178.3±6.0	109.3±5.0	284.6±9.1	181.5±5.8	105.8±4.1	290.5±8.0	180.5±4.3	106.9±3.2	288.5±6.1
SEPARATE (df=4/5)	197.9±4.4	115.9±5.1	314.5±7.4	190.7±5.6	109.6±4.4	300.6±7.6	192.8±4.0	111.7±3.4	305.2±5.7
ABS-AC (df=5/5)	189.1±7.0	127.8±5.7	308.2±9.8	195.3±5.4	124.8±4.5	314.8±7.4	193.2±4.3	125.8±3.5	312.6±5.9

Supplementary Table 1: BICs as a function of the dataset used for parameter optimization (Learning sessions, Transfer test or Both) and the computational model. nt: number of trials; df: degree of freedom.

	Experiment 1 (N=20)			Experiment 2 (N=40)			Both experiments (N=60)		
	Learning sessions (nt=160)	Transfer test (nt=112)	Both (nt=272)	Learning sessions (nt=160)	Transfer test (nt=112)	Both (nt=272)	Learning sessions (nt=160)	Transfer test (nt=112)	Both (nt=272)
ABSOLUTE (df=2/3)	179.8±5.9	113.6±5.7	295.1±9.4	190.6±4.7	125.2±4.2	324.2±6.4	187.0±3.7	121.3±3.4	315.5±5.5
RELATIVE (df=2/3)	193.6±4.6	136.5±5.1	329.3±8.4	184.7±5.6	119.0±4.1	303.6±7.6	187.7±4.0	124.8±3.4	312.2±6.0
HYBRID (df=3/4)	178.3±6.0	107.5±5.1	284.6±9.1	181.0±5.7	103.2±4.0	288.2±8.0	180.1±4.3	104.6±3.2	287.0±6.1
POLICY (df=2/3)	185.4±6.9	121.3±5.8	308.0±11.8	189.5±4.8	135.5±3.7	333.0±6.4	188.1±3.9	130.7±3.3	323.3±5.9
UTILITY (df=3/4)	173.9±6.5	107.4±6.3	282.2±10.8	182.8±5.5	122.2±4.4	308.4±7.1	179.9±4.3	117.3±3.7	299.6±6.1
SEPARATE (df=4/5)	196.7±4.4	115.0±5.3	312.5±7.7	189.2±5.4	107.7±4.3	299.4±7.4	191.7±3.9	110.4±3.3	303.7±5.6
ABS-AC (df=5/5)	183.3±7.3	127.7±5.7	300.7±10.2	193.0±5.3	120.1±4.5	312.5±7.2	190.3±4.2	122.8±3.6	309.1±5.9

Supplementary Table 2: BICs as a function of the dataset used for parameter optimization (Learning sessions, Transfer test or Both) and the computational model using multiple starting points (5 different random initializations per parameter, model and subject). nt: number of trials; df: degree of freedom.
